# Chimeric human opsins as optogenetic light sensitisers

**DOI:** 10.1242/jeb.240580

**Published:** 2021-07-14

**Authors:** Doron G. Hickey, Wayne I. L. Davies, Steven Hughes, Jessica Rodgers, Navamayooran Thavanesan, Robert E. MacLaren, Mark W. Hankins

**Affiliations:** 1 Nuffield Laboratory of Ophthalmology, University of Oxford, Oxford, OX1 3QU, UK; 2 The Royal Victorian Eye and Ear Hospital, Melbourne, VIC 3002, Australia; 3 Umeå Centre for Molecular Medicine, Umeå University, Umeå, S-90187, Sweden; 4 School of Life Sciences, College of Science, Health and Engineering, La Trobe University, Melbourne, VIC 3086, Australia; 5 Sleep and Circadian Neuroscience Institute, University of Oxford, Oxford, OX1 3QU, UK; 6 Division of Neuroscience and Experimental Psychology, University of Manchester, Manchester, M13 9PT, UK; 7 Oxford Eye Hospital, Oxford University Hospitals NHS Foundation Trust and Oxford NIHR Biomedical Research Centre, Oxford, OX3 9DU, UK

**Keywords:** Retina, Opsin, Chimera, G protein, Optogenetics, Phototransduction

## Abstract

Human opsin-based photopigments have great potential as light-sensitisers, but their requirement for phototransduction cascade-specific second messenger proteins may restrict their functionality in non-native cell types. In this study, eight chimeric human opsins were generated consisting of a backbone of either a rhodopsin (RHO) or long-wavelength-sensitive (LWS) opsin and intracellular domains from G_q/11_-coupled human melanopsin. Rhodopsin/melanopsin chimeric opsins coupled to both G_i_ and G_q/11_ pathways. Greater substitution of the intracellular surface with corresponding melanopsin domains generally showed greater G_q/11_ activity with a decrease in G_i_ activation. Unlike melanopsin, rhodopsin and rhodopsin/melanopsin chimeras were dependent upon exogenous chromophore to function. By contrast, wild-type LWS opsin and LWS opsin/melanopsin chimeras showed only weak G_i_ activation in response to light, whilst G_q/11_ pathway activation was not detected. Immunocytochemistry (ICC) demonstrated that chimeric opsins with more intracellular domains of melanopsin were less likely to be trafficked to the plasma membrane. This study demonstrates the importance of G_α_ coupling efficiency to the speed of cellular responses and created human opsins with a unique combination of properties to expand the range of customised optogenetic biotools for basic research and translational therapies.

## Abstract (precis)

**Summary:** Combining different domains of human visual opsins and melanopsin creates functionally unique chimeric opsins with potential optogenetic applications.

## INTRODUCTION

Optogenetics is the use of light-sensitive molecules to confer photosensitivity to cells that are not intrinsically photoreceptive, enabling the manipulation of cellular function by a light stimulus (Yizhar et al., 2011). A potential application of optogenetics is vision restoration via gene therapy.

Several photosensitive molecules have been investigated as optogenetic tools for vision restoration, including human rhodopsin, melanopsin and medium wavelength opsin (Lin et al., 2008; van Wyk et al., 2015; Gaub et al., 2015; Cehajic-Kapetanovic et al., 2015; Berry et al., 2019). Although these studies have shown some success, the light sensitivity and second messenger coupling of these photosensitive molecules are limited. There are clear advantages of coupling to a ubiquitous signalling pathway to facilitate amplification and thus increase light sensitivity.

As native opsins do not have all the ideal properties of an optogenetic biotool, a number of studies have generated chimeric opsins that combine the desired functional properties of the individual proteins. Like all G protein-coupled receptors (GPCRs), rhodopsin (RHO), long-wavelength-sensitive (LWS) cone opsin and melanopsin have a common structure that includes seven transmembrane α-helices, linked by intracellular and extracellular loops, and extracellular N and intracellular C termini (Oldham and Hamm, 2008). There are multiple G protein signalling pathways, and a GPCR's ability to activate each pathway is determined by its affinity for each G protein. G proteins are heterotrimeric proteins consisting of three subunits: α, β and γ. It is the α subunit that largely defines the properties of each G protein (Arshavsky et al., 2002), which, based on structural and functional similarities, is divided into four classes: G_i/o_, G_s_, G__q/11_ _and G_12/13_ (Wilkie et al., 1992). It is the intracellular loops (ICLs), as part of the intracellular surface (ICS), that are most critical for determining G protein selectivity (Kim et al., 2005; Bailes et al., 2012; Airan et al., 2009). The structural homology of GPCRs can permit protein domains from one GPCR to be substituted with the corresponding regions from another to confer some of the functional properties of the donor protein, as first demonstrated on adrenergic receptors (Cotecchia et al., 1990; Kobilka et al., 1988). To date, opsin-based chimeras where the opsins are very closely related have had the greatest success in generating viable proteins that differ in their spectral properties (Asenjo et al., 1994; Shi et al., 2001), but not necessarily in their signalling activation properties (McClements et al., 2013a,b; Matsushita et al., 2014). Viable chimeric proteins between distantly related GPCRs have also been created (Kim et al., 2005; Bailes et al., 2012; Airan et al., 2009). These studies established that replacing the ICS can result in rhodopsin signalling via a non-native pathway.

In the present study, chimeras of human melanopsin with domains derived from human visual opsins, namely rhodopsin and LWS opsin, were generated to investigate their utility as G_q/11_-signalling optogenetic biotools. Using human opsin-derived biotools facilitates the translation of therapeutic applications to the clinic, as patients will have immune tolerance to human-derived proteins, thereby reducing the risk of rejection.

Rhodopsin and LWS opsin were selected because of their light sensitivity, rapid response to light, distinct spectral sensitivities and the fact that they are relatively well characterised. Melanopsin was selected because it couples to the ubiquitous excitatory G_q/11_ pathway (Melyan et al., 2005; Graham et al., 2008; Bailes and Lucas, 2013), meaning that the opsin/melanopsin chimeras could be utilised to light-sensitise retinal cell types, such as bipolar cells, that do not natively express visual opsins. G_q/11_ second messengers are ubiquitous in the retina (Hughes et al., 2015) and are found throughout the body, with at least one or both of G_q_ and G_11_ found in every cell type screened so far (Hughes et al., 2015; Wilkie et al., 1991; Strathmann and Simon, 1990; Hubbard and Hepler, 2006). By contrast, rhodopsin does not activate the G_q/11_ pathway in mammals (Terakita et al., 1998) and the native G protein partner of rhodopsin, the rod isoform of transducin (G_t[rod]_), is exclusively expressed in rod photoreceptors (Lerea et al., 1986). Nonetheless, rhodopsin can couple to other G_α_ subunits of the G_i/o_ family to which it belongs both *in vitro* (Bailes and Lucas, 2013; Kanaho et al., 1984) and *in vivo* (Gutierrez et al., 2011; Li et al., 2005), so rhodopsin-based chimeras expressed outside of photoreceptors should maintain some level of functionality in the presence of a supply of *cis-*retinal chromophore.

**Fig. 1.**
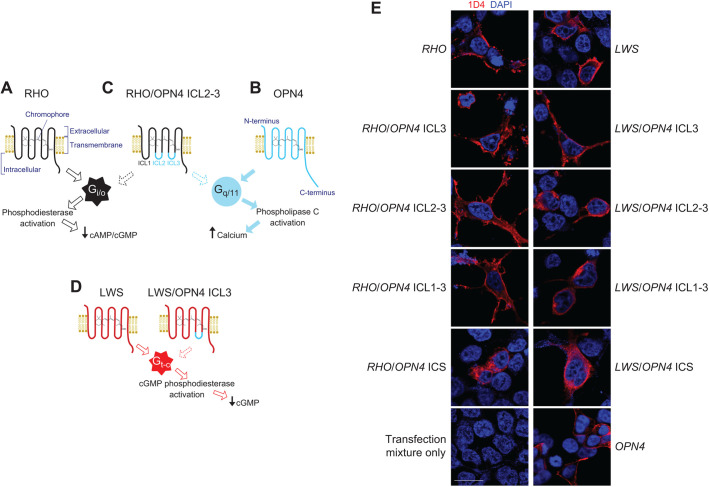
**Human visual opsin/melanopsin chimeric G protein-coupled receptors (GPCRs).** (A) Human rhodopsin (RHO) activates G protein partners of the G_i/o_ family, which activates phosphodiesterase enzymes, resulting in a reduction in cyclic nucleotide second messenger concentration. (B) Melanopsin (OPN4) activates the G_q/11_ G protein signalling cascade, leading to phospholipase C activation and intracellular Ca^2+^ release. Chimeric opsins consisting of a backbone of either a (C) rhodopsin (RHO, black) or (D) long-wavelength-sensitive (LWS, red) opsin and intracellular domains from melanopsin (OPN4, cyan) were created and their G protein coupling properties were tested [RHO/OPN4 ICL2-3 (C) and LWS/OPN4 ICL3 (D) shown as examples]. LWS opsin activates cone transducin, G_t[cone]_, which activates cGMP phosphodiesterase, resulting in a reduction in cyclic guanosine monophosphate (cGMP) second messenger concentration. (E) Immunocytochemistry (ICC) of HEK293T cells expressing chimeric and wild-type opsins. Wild-type rhodopsin, LWS opsin, melanopsin and chimeric opsin plasmids (all 1D4 tagged) were immunolabelled with an antibody against the 1D4 epitope. ICS, intracellular surface. Scale bar: 20 μm.

Specifically, the present study generated two groups of human chimeric opsins where the degree of domain swapping was varied (Fig. 1). The first group combined the transmembrane and extracellular domains of rhodopsin with a variable amount of ICS that was substituted by corresponding melanopsin regions. It was hypothesised that these chimeric opsins would be highly light-sensitive, be switchable by requiring an exogenous chromophore supply (properties derived from rhodopsin), while also coupling to and activating the ubiquitous G_q/11_ protein pathway (a property of melanopsin).

The second group of chimeras was similarly constructed, but used LWS cone opsin (λ_max_ 552–562 nm; McClements et al., 2013b; Merbs and Nathans, 1992) domains, instead of those of rhodopsin (λ_max_ ∼500 nm; Crescitelli and Dartnall, 1953), with the intention of creating a red-shifted G_q/11_ coupled opsin with potentially faster kinetics. LWS opsin natively activates a cone-specific form of transducin, G_t[cone]_, and has been shown to couple to G_t[rod]_ *in vitro* but not *in vivo* (Lerea et al., 1986; Imamoto et al., 2013). Coupling of LWS opsin to the G_i/o_ family has been reported (Ballister et al., 2018). In general, cones have faster onset and offset kinetics and are more bleach resistant than rods (Imamoto et al., 2013), which provides the potential for visual opsin/melanopsin chimeras that are functionally distinct and appealing as optogenetic tools for use in multiple biological systems, such as vision-restoring therapies.

This study demonstrates that human LWS opsin and LWS-derived chimeric opsins weakly activate the G_i_, but not the G_q/11_, intracellular pathway in human embryonic kidney (HEK293T) cells. In contrast, rhodopsin/melanopsin chimeric human opsins are able to activate both G_q/11_ and G_i_ intracellular pathways and require exogenous chromophore for normal functioning.

## MATERIALS AND METHODS

### Generation of chimeric opsin constructs

The coding regions of human rhodopsin (GenBank accession number: NM000539), melanopsin (*OPN4*; NM033282) and LWS opsin (NM020061) were cloned into the pMT4 mammalian expression vector, as previously described (Davies et al., 2012). Human opsin intracellular/transmembrane boundaries were based on the crystal structure of bovine rhodopsin (Palczewski, 2000), as used in previous studies (Davies et al., 2007a) (Figs S1 and S2). Primers were designed to gene regions that crossed these boundaries and the SPLICE technique (Davies et al., 2007b) was used to amplify and generate four *RHO*/*OPN4* chimeras and four *LWS*/*OPN4* chimeric opsins. The plasmid encoding a chimeric bovine rhodopsin/human α_1a_-adrenergic receptor, pcDNA3.1/opto-a1AR-EYFP (Airan et al., 2009), was obtained from a plasmid repository (Addgene).

### Cells and transient transfection

HEK293T cells (ATCC) were maintained in Dulbecco's modified eagle medium (DMEM) (Sigma-Aldrich), containing 10% fetal bovine serum (FBS) (Sigma-Aldrich), 1% L-glutamine (Sigma-Aldrich) and 1% penicillin/streptomycin solution (Sigma-Aldrich) in a humidified incubator at 37°C with 5% CO_2_. HEK293T cells were transfected using GeneJuice transfection reagent (Merck Millipore) according to the manufacturer's instructions.

### Immunocytochemistry

Immunocytochemistry (ICC) was performed using standard techniques. Briefly, 48 h post-transfection HEK293T cells were fixed with methanol-free 4% formaldehyde (Thermo Fisher Scientific) in PBS for 10 min at room temperature. Transfected cells were washed three times with 0.05% Tween-20 in PBS (PBS-T), permeabilised with 0.2% Triton X-100 in PBS for 5 min and blocked with 5% donkey serum (Sigma-Aldrich) in PBS-T for 30 min. Cells were incubated for 1 h with primary rabbit polyclonal anti-rhodopsin (ab3424, http://www.abcam.com/rhodopsin-antibody-ab3424.html) and mouse monoclonal anti-1D4 antibodies (gift from Dr Jill Cowing, Institute of Ophthalmology, University College London), both diluted 1:1000 in 1% donkey serum in PBS-T. Cells were washed five times with PBS-T. Cells were incubated for 30 min with Alexa-488 and Alexa-568 conjugated secondary antibodies (A10037 and A21206, Life Technologies), 1:200 with 1% donkey serum in PBS-T before five washes with PBS-T. All steps were carried out at room temperature. Coverslips were mounted with ProLong Gold with DAPI (Life Technologies), left in the dark overnight and then stored at 4°C. Fluorescent images were acquired using an inverted confocal microscope (LSM 710, Carl Zeiss).

### Live cell cyclic adenosine monophosphate (cAMP) assay

Intracellular cAMP concentrations were assayed using the bioluminescence cAMP reporter pGloSensor-22F (Promega) using methods previously described (Bailes and Lucas, 2013). HEK293T cells were seeded into white-walled 96-well plates (Sarstedt) containing complete CO_2_-independent medium (Gibco) and maintained in a humidified incubator at 37°C with 5% CO_2_. For transfection, each well received 0.3 µl of GeneJuice, 50 ng of GloSensor-22F and 50 ng of an opsin plasmid. Forty-eight hours after transfection, cells were loaded with 20 µmol l^−1^ 9-*cis* retinal (Sigma-Aldrich) and 4 mmol l^−1^ beetle luciferin (Promega) under dim red light conditions and incubated at room temperature in the dark for at least 2 h. 9-*cis* retinal was chosen over 11-*cis* retinal because of its commercial availability, and due to both having comparable properties for opsin activation (Davies et al., 2011).

Recording of luminance values was performed using a FLUOStar Omega fluorescence plate reader (BMG Labtech) and Omega software (3.00 R2, BMG Labtech). Imaging was performed at 25–27°C. Bioluminescence values were collected sequentially from each well every 30 s using 1 s collection times. The following protocol was used: (1) baseline for 5 cycles of bioluminescence measurement; (2) plate ejection for 35 s, where forskolin (final concentration, 2 µmol l^−1^; Sigma-Aldrich) was added to each well to elevate cAMP levels; (3) measurement of the forskolin bioluminescent response for 14 cycles; (4) application of light stimuli that consisted of 100 Hz flashes for 2 s from an internal xenon flash lamp passed through either a 485 or 544 nm bandpass filter (10 nm bandwidth; BMG Labtech); and (5) final measurement of the bioluminescent response for 35 cycles. Data for each well were normalised to the first value of the light response phase of the protocol (i.e. step 4). Results from four technical replicates on a given plate were averaged for each biological replicate.

### Live cell calcium assays

Levels of intracellular calcium (Ca^2+^) were assayed using Fluo-4 AM ester (Thermo Fisher) fluorescent Ca^2+^ indicator dye or, when repeat light stimulation was required, a genetically encoded Ca^2+^ indicator, GCaMP6f (Chen et al., 2013). HEK293T cells in black-walled 96-well plates in complete CO_2_-independent medium were transfected with opsin plasmids (and, where stated, GCaMP6f plasmid). After 48 h post transfection, cells were loaded with Fluo-4 AM (final concentration 5 µmol l^−1^), probenecid (1.25 mmol l^−1^) (Thermo Fisher) and, where stated, 9-*cis* retinal (20 µmol l^−1^) and incubated for 45 min at 37°C in darkness prior to imaging. For cells transfected with GCaMP6f, cells were imaged before and after incubation with 20 µmol l^−1^ 9-*cis* retinal for 45 min (see below). All incubation steps were conducted under dim red light.

Light-induced changes in intracellular Ca^2+^ levels were quantified using a FLUOStar Omega fluorescence plate reader and Omega software (3.00 R2, BMG Labtech). Imaging was performed at 25–27°C. For each well, individual data points were collected by averaging values from 200 flashes (100 Hz) of 485 nm light, with data points collected every 2 s for 2 min. The light flashes were sufficient and necessary to stimulate light responses. Each well was imaged sequentially. Data were exported from MARS data analysis software (BMG Labtech). Data for each well were normalised to the first value from that well, and data from four wells per plate were averaged (technical replicates).

### Statistical analysis

Statistical analyses were performed using Prism (6.0 h, GraphPad). The area under the curve (AUC) function was used to calculate both the AUC and the time at which the peak relative fluorescence value was reached. The duration over which the AUC was calculated was the time from light stimulus to the completion of the assay. When comparing one independent variable with more than two conditions and a single dependent variable, an ordinary one-way ANOVA was applied. *Post hoc* tests were conducted with Bonferroni's multiple comparisons tests. Where possible, sample groups were all compared with a single control group; otherwise, all groups were compared with each other. Graphical data are presented as means±s.e.m. α=0.05 for all tests.

## RESULTS

### Generating chimeric opsins

Eight novel chimeric constructs were generated based on the transmembrane and extracellular domains of either human rhodopsin or LWS cone opsin and containing varying number of intracellular domains from human melanopsin (Fig. 1; Figs S1 and S2). The four rhodopsin/melanopsin chimeric opsins were (corresponding LWS/melanopsin chimeric opsins names in parentheses): (1) rhodopsin with the third ICL of melanopsin, designated as *RHO*/*OPN4* ICL3 (*LWS*/*OPN4* ICL3); (2) rhodopsin with the second and third ICLs of melanopsin, *RHO*/*OPN4* ICL2-3 (*LWS*/*OPN4* ICL2-3); (3) rhodopsin with the first, second and third ICLs of melanopsin, *RHO*/*OPN4* ICL1-3 (*LWS*/*OPN4* ICL1-3); and (4) rhodopsin with the entire ICS (that is, the three ICLs and the intracellular C-terminal sequence) of melanopsin, *RHO*/*OPN4* ICS (*LWS*/*OPN4* ICS). All constructs contained a 1D4 epitope (originally from rhodopsin) located at their C terminus.

### Subcellular localisation of chimeric opsins

HEK293T cells transfected with the chimeras, human rhodopsin, LWS opsin or melanopsin (all of which contain C-terminal 1D4 epitope tags) were immunolabelled with two different anti-rhodopsin antibodies (Fig. 1E; Figs S3 and S4). Using a mouse monoclonal antibody shown to recognise the 1D4 epitope (MacKenzie et al., 1984), cells transfected with *RHO*, *RHO*/*OPN4* ICL3, *RHO*/*OPN4* ICL2-3 and *RHO*/*OPN4* ICL1-3 showed a strong labelling at the plasma membrane and at distal processes. *RHO*/*OPN4* ICS transfected cells, which had a more rounded appearance, showed more even distribution of 1D4 staining throughout the cytoplasm (Fig. 1E). Immunolabelling with a rabbit polyclonal antibody raised against a peptide sequence corresponding to the distal C terminus of bovine rhodopsin (which contains the 1D4 epitope) also showed strong labelling at the plasma membrane for cells transfected with *RHO* and *RHO/OPN4* ICL3 (Fig. S3). However, for cells transfected with *RHO*/*OPN4* ICL2-3, *RHO*/*OPN4* ICL1-3 and *RHO*/*OPN4* ICS, the signal from this polyclonal antibody was relatively weak at the plasma membrane but strong at distinct foci within the cytoplasm.

A similar pattern of staining was observed for LWS opsin and LWS opsin/melanopsin chimeric opsins. Cells transfected with *LWS* opsin and *LWS*/*OPN4* ICL3 showed strong labelling at the plasma membrane using both antibodies, while the labelling from both antibodies was weaker at the plasma membrane as the number of intracellular domains of melanopsin increased (Fig. S4). In melanopsin-transfected cells, the monoclonal antibody against 1D4 gave strong signal at the plasma membrane (Fig. 1E).

### Functional assessment of rhodopsin/melanopsin chimeras

**Fig. 2.**
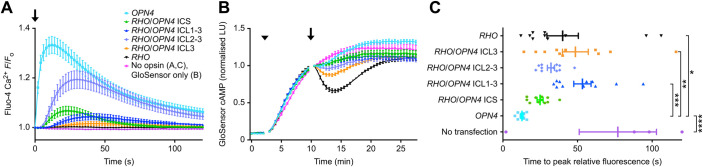
***In vitro* G_q/11_ and G_i_ function of rhodopsin/melanopsin chimeric opsins and wild-type rhodopsin and melanopsin.** (A) In a plate reader-based assay of G_q/11_ activity, transfected HEK293T cells were loaded with Fluo-4 dye before being illuminated with 485 nm light (arrow). The relative fluorescence unit value (*F*) was normalised to the value of the first reading (*F*_o_) (*n*=11). (B) The ability of rhodopsin/melanopsin chimeric opsins, wild-type rhodopsin and melanopsin to couple to the G_i_ pathway was assayed using GloSensor, a cAMP biosensor. Baseline bioluminescence was measured before forskolin was added (arrowhead) to raise the baseline level of cAMP. Cells were illuminated with 485 nm light (arrow) (mean±s.e.m.; *n*=8). (C) The time to peak relative fluorescence showed statistically significant differences between the groups (ordinary one-way ANOVA, *P<*0.0001, *n*=11). *P<0.05, **P<0.01, ***P<0.001, ****P<0.0001. ICL, intracellular loop; ICS, intracellular surface; LU, luminescence units.

The cellular functions of the rhodopsin/melanopsin chimeric opsins were assessed using *in vitro* assays of G_q/11_ and G_i_ signalling cascades, for which wild-type human melanopsin and human rhodopsin served as positive controls, respectively. G_q/11_ activation was assessed using the fluorescent Ca^2+^ indicator Fluo-4. Melanopsin-transfected HEK293T cells produced a rapid increase in intracellular Ca^2+^ levels following illumination with 485 nm light (Fig. 2A). Cells transiently transfected with human rhodopsin showed a clear light-dependent reduction in cAMP-dependent bioluminescence, while human melanopsin produced a small decrease in bioluminescence relative to a no-opsin control (Fig. 2B).

Rhodopsin/melanopsin chimeras displayed properties that were intermediate between that of rhodopsin and melanopsin. As more of the intracellular rhodopsin protein surface was substituted with the corresponding region of melanopsin, G_q/11_ activation by the chimeric pigments generally increased (Fig. 2A) and their ability to activate G_i_ decreased (Fig. 2B). However, none of the chimeric opsins functioned as efficiently as the corresponding wild-type melanopsin protein.

*RHO*/*OPN4* ICL3 produced a cAMP response that was the largest of all the chimeras tested, but this was still only ∼50% of that evoked by wild-type rhodopsin, as measured by the local nadir of cAMP-dependent bioluminescence following light stimulation (i.e. at ∼13–14 min) (Fig. 2B). Conversely, *RHO*/*OPN4* ICL3 produced the smallest Ca^2+^ responses of all chimeras tested, producing only a small and slow increase in signal relative to rhodopsin and no-opsin controls (Fig. 2A). *RHO*/*OPN4* ICL2-3, containing both the second and third intracellular loops of melanopsin, showed moderate activity in both G_i_ and G_q/11_ assays. *RHO*/*OPN4* ICL2-3 produced a decrease in cAMP-dependent bioluminescence that was notably greater and more sustained than that of melanopsin, but less than that of *RHO*/*OPN4* ICL3 (Fig. 2B). *RHO*/*OPN4* ICL2-3 produced the largest mean peak Ca^2+^ signal of all four rhodopsin-based chimeras tested (Fig. 2A). *RHO*/*OPN4* ICL1-3 and *RHO*/*OPN4* ICS produced similar results for both cAMP and Ca^2+^ assays: both slightly reduced intracellular cAMP following illumination with 485 nm light, and both produced Ca^2+^ responses larger than those exhibited by no-opsin and rhodopsin controls and intermediate between those of *RHO*/*OPN4* ICL3 and *RHO*/*OPN4* ICL2-3.

The AUC following light stimulus in the cAMP assay (10.5–27.5 min) was used to quantify the magnitude of G_i_ activation (Everett and Cooper, 2013) (Fig. S5A). There was a statistically significant difference between rhodopsin/melanopsin chimeras, wild-type rhodopsin, wild-type melanopsin and the no-opsin group (ordinary one-way ANOVA, *F*_6,49_=11.8, *P*<0.0001, *n*=8). *Post hoc* testing, with Bonferroni correction, found statistically significant smaller AUC values for the following groups relative to the no-opsin group (20.3±0.8 units): rhodopsin (15.5±0.3; *P*<0.0001), *RHO*/*OPN4* ICL3 (17.5±0.3; *P*<0.01) and *RHO*/*OPN4* ICL2-3 (18.0±0.6; *P*<0.05). AUC values from the intracellular Ca^2+^ assay were utilised to quantify the magnitude of G_q/11_ activation and showed a statistically significant difference between all groups (ordinary one-way ANOVA, *F*_6,70_=28.2, *P*<0.0001, *n*=11; Fig. S5B). *Post hoc* testing, with Bonferroni correction, found statistically significant greater AUC values for the following groups relative to the no-opsin group (0.72±0.13 units): *RHO*/*OPN4* ICL2-3 (12.87±4.19; *P*<0.0001) and *OPN4* (19.72±4.03; *P*<0.0001).

The relative time to peak fluorescence showed statistically significant differences between all groups (ordinary one-way ANOVA, *F*_6,62_=6.4, *P*<0.0001; *n*=11; Fig. 2C). *Post hoc* testing, with Bonferroni correction, showed statistically significant greater time to peak values for the following groups compared with melanopsin (12.7±0.8 s): rhodopsin (40.0±10.5 s; *P*<0.05), *RHO*/*OPN4* ICL3 (48.5±8.6 s; *P*<0.01), *RHO*/*OPN4* ICL1-3 (53.3±5.8 s; *P*<0.001) and the no-opsin control (77.0±25.9 s; *P*<0.0001).

### Rhodopsin/melanopsin chimeric opsins require exogenous chromophore

Rhodopsin and all rhodopsin/melanopsin chimeric photopigments were dependent on exogenous 9-*cis* retinal for biological function. In the absence of 9-*cis* retinal chromophore, transfected cells did not display the characteristic inhibition of cAMP-dependent bioluminescence (Fig. S5C). However, the addition of 9-*cis* retinal to the cells led to the return of the rhodopsin and *RHO*/*OPN4* ICL3 light-induced decrease in signal (Fig. S5D). Similarly, in the Ca^2+^ assay, no light-dependent increase in signal was detected from any of the rhodopsin/melanopsin chimeras unless cells were first incubated with 9-*cis* retinal (Fig. S5E,F). By contrast, there was no distinguishable difference in the response characteristics of wild-type melanopsin with or without added 9-*cis* retinal.

### Rhodopsin/melanopsin chimeras compared with Opto-α_1_AR chimeric opsin

The G protein activation characteristics of the rhodopsin/melanopsin chimeras were compared with those of another chimeric opsin, Opto-α_1_AR (Airan et al., 2009). Opto-α_1_AR consists of the transmembrane and extracellular domains of bovine rhodopsin and the ICS (intracellular loops and C terminus) of human α_1a_-adrenergic receptor, and has been shown to couple to the G_q/11_ second messenger signalling pathway (Airan et al., 2009). In the Ca^2+^ assay, Opto-α_1_AR produced a moderate response that was slower to reach a lower peak than either *RHO*/*OPN4* ICL2-3 or *RHO*/*OPN4* ICS (Fig. S5F). However, the Opto-α_1_AR Ca^2+^ response was greater than that of either *RHO*/*OPN4* ICL3 or *RHO*/*OPN4* ICL1-3 (Fig. S5F). The offset kinetics of the Opto-α_1_AR Ca^2+^ response were notably different to those of rhodopsin/melanopsin chimeras: cells transfected with Opto-α_1_AR showed an increase in Ca^2+^ signal that continued throughout the 2 min recording period, while responses from cells expressing rhodopsin/melanopsin chimeras reached maximal responses by ∼45 s and then decreased towards baseline values. In the cAMP assay, Opto-α_1_AR-transfected cells did not show any light-dependent change in bioluminescence, showing responses similar to those of no-opsin controls (Fig. S5D). As with rhodopsin/melanopsin chimeras, Opto-α_1_AR only produced a measurable increase in Ca^2+^ levels following the addition of 9-*cis* retinal (Fig. S5E,F).

### Functional assessment of LWS/OPN4 chimeric opsins

**Fig. 3.**
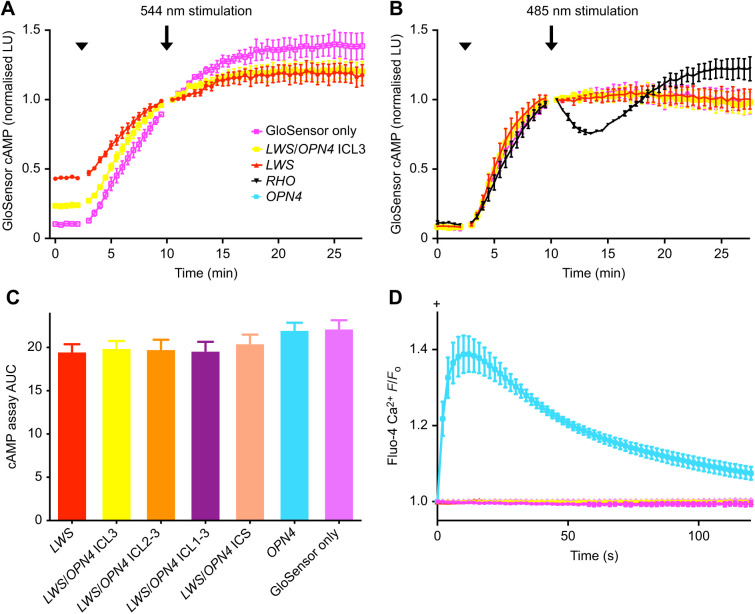
***In vitro* G_i_ and G_q/11_ function of *LWS*/melanopsin chimeric opsins and wild-type *LWS* opsin and melanopsin.** (A,B) The ability of wild-type LWS opsin and LWS opsin/melanopsin chimeric opsins (just LWS/OPN4 ICL3 is shown for clarity) to couple to the G_i_ pathway was assayed using the GloSensor cAMP reporter. Forskolin was added (arrowhead) to raise baseline levels of cAMP. Cells were then illuminated with (A) 544 nm or (B) 485 nm light at the 10 min time point (arrow). Illumination at 544 nm induced a change in G_i_ activity in *LWS* opsin-transfected cells and a smaller change in *LWS*/*OPN4* ICL3-transfected cells, compared with cells transfected with only the GloSensor plasmid (mean±s.e.m.; *n*=4). *LWS* opsin- and *LWS*/*OPN4* ICL3-transfected cells did not produce any detectable change in G_i_ activity, relative to GloSensor only cells, in response to 485 nm light. (C) Analysis of the area under the curve (AUC) of 544 nm cAMP assay data showed a statistically non-significant trend of increased area from wild-type LWS opsin to the GloSensor only negative control group (ordinary one-way ANOVA, *P<*0.142). (D) Monitoring of G_q/11_ activity using a fluorescent Ca^2+^ indicator dye, Fluo-4, shows that neither LWS opsin nor the LWS opsin/melanopsin chimeric opsins showed a Ca^2+^ rise in response to 485 nm light (*n*=3). *F*/*F*_o_, fluorescence intensity relative to baseline fluorescence; ICL, intracellular loop; ICS, intracellular surface; LU, luminescence units; arrowhead, forskolin added; arrow, light stimulation.

Cells expressing LWS opsin showed small light-induced changes in cAMP-dependent bioluminescence, relative to no-opsin control cells, when stimulated with 544 nm light (Fig. 3A; Fig. S6A). Such differences between LWS opsin and no-opsin control cells were not seen with a 485 nm light stimulus (Fig. 3B; Fig. S6B). With increased substitution of the ICS for corresponding domains of melanopsin, the cAMP response in response to 544 nm light decreased, as quantified by analysis of the AUC (ordinary one-way ANOVA, *F*_6,21_=1.40, *P*=0.262, *n*=4) (Fig. 3C). Unlike rhodopsin/melanopsin chimeric opsins, neither LWS opsin nor any of the LWS opsin/OPN4 chimeras produced a light-dependent change in intracellular Ca^2+^ levels (Fig. 3D).

## DISCUSSION

### Rhodopsin/melanopsin chimeras can couple to two distinct G protein pathways

This study has demonstrated that chimeras consisting of rhodopsin extracellular and transmembrane regions with melanopsin intracellular domains require exogenous chromophore and signal via both the G_q/11_ and G_i_ pathways.

Substituting the third ICL of rhodopsin with that of melanopsin alone was sufficient to produce small responses in a Ca^2+^ assay of G_q/11_ activation, where wild-type rhodopsin showed no such response. This modification resulted in a reduction in G_i_ activity when tested using the cAMP assay. Replacing the second and third loops resulted in a greater increase in G_q/11_ activation and decreased G_i_ activity, demonstrating that the third ICL together with the second ICL is critical for the activation of G protein signalling pathways (Scheerer et al., 2008), which has been suggested for bovine rhodopsin (Yamashita et al., 2000; Terakita et al., 2002), murine melanopsin (van Wyk et al., 2015) and the β2 adrenergic receptor (Kim et al., 2005).

Additional alterations to the rhodopsin ICS (e.g. by including the first intracellular melanopsin domain and/or the melanopsin C terminus) led to reduced activation of both G_i_ and G_q/11_ signalling cascades when compared with substitution of only the second and third ICL. These data suggest that the first intracellular domain and the C terminus may also contribute to (and potentially inhibit) G protein binding. In all cases, the rhodopsin/melanopsin chimeras showed a rapid onset of G_q/11_ activation, but with slower kinetics than wild-type melanopsin.

These data indicate that an opsin's efficiency of activation of G_α_ subunits is critically important to defining the speed of cellular responses to light stimuli, rather than being a product of the intrinsic speed of the opsin itself. Rhodopsin is known to mediate very fast cellular responses in rod cells (Korenbrot, 2012), but modifying rhodopsin by the substitution of the third ICL to create RHO/OPN4 IL3 created an opsin that coupled to the G_q/11_ pathway to a greater extent than wild-type rhodopsin, but was slow to reach its peak level of Ca^2+^ signal. With the substitution of more of the intracellular domains of rhodopsin for those of melanopsin, the time taken to reach a peak in the Ca^2+^-dependent signal is generally reduced. This suggests that as G_q/11_ activation becomes more efficient (with more melanopsin-derived intracellular domains) the peak Ca^2+^ response becomes faster. The functional activity of the rhodopsin/melanopsin chimeras correlate with the immunocytochemistry results: wild-type rhodopsin and all rhodopsin/melanopsin chimeric opsins, except RHO/OPN4 ICS, were largely localised at the plasma membrane. Partial reduction in plasma membrane trafficking might explain why both G_q/11_ and G_i_ activation by RHO/OPN4 ICS were similar to that by RHO/OPN4 ICL1-3.

By comparing the functional output after transfection with a standardised mass of plasmid, our protocol was designed to account for differences in both the quantity of functional protein and the efficacy of each protein for cAMP and Ca^2+^ signalling. This protocol reflects the clinical scenario of delivering a standard dose of gene therapy vector to a patient and measuring the functional (visual) gains. Protein quantification methods could assist in differentiating the quantities of folded versus misfolded protein, to further understand what contribution non-functional opsins made to the net functional effect.

With increased intracellular domain substitution, a divergence in anti-rhodopsin staining with monoclonal and polyclonal antibodies was observed, with a punctate labelling pattern observed using the polyclonal antibody that suggested the formation of aggresomes – specialised structures that are formed when the proteolytic machinery of a cell is saturated by misfolded protein (Saliba et al., 2002). The different labelling pattern of the antibodies may be explained by potential epitope specificity differences.

Although the conformational epitopes recognised by the polyclonal antibody have not been characterised, the antibody was raised against a synthetic peptide antigen (Abcam, 2017) that neither has the post-translational modifications that are common on RHO (Zhang et al., 1997) nor is specific to the conformation of native RHO. By contrast, the 1D4 monoclonal antibody, which was initially raised against bleached bovine rod outer segment disk membranes (Molday and MacKenzie, 1983), has been shown to have a higher preference for the native structural conformation of RHO compared with peptide antigens (MacKenzie et al., 1984). Having been processed by the cellular machinery, 1D4-tagged chimeric opsins are likely to have the post-translational modifications that create the conformational epitopes to which 1D4 has a high affinity.

Taking the different immunocytochemistry results into consideration suggests that the polyclonal antibody may have more affinity for the misfolded form of the chimeric opsin compared with the monoclonal antibody. These results suggest that this dual-labelling technique could provide additional information regarding opsin processing and function compared with single labelling.

### Distinct chromophore requirements of wild-type and chimeric opsins

The chromophore requirements of melanopsin differed from that of rhodopsin and the rhodopsin/melanopsin chimeric opsins. Wild-type melanopsin-transfected HEK293T cells had a consistent Ca^2+^ response regardless of whether exogenous 9-*cis* retinal was added. In contrast, no Ca^2+^ or cAMP response was elicited from rhodopsin or rhodopsin/melanopsin chimeric opsins without the addition of 9-*cis* retinal. These data lend support to the evidence that melanopsin is bistable (Melyan et al., 2005; Matsuyama et al., 2012) (or perhaps tristable; Emanuel and Do, 2015) and is therefore able to utilise other chromophores, such as all-*trans* retinal, that cannot form a stable association with visual opsins. Human embryonic kidney cells are able to convert all-*trans* retinal to *cis* retinal, but this process takes hours, so would not account for the differences recorded (Brueggemann and Sullivan, 2002). Having a G_q/11_-coupled, chromophore-dependent opsin ensures that the chimeric opsin will only be active in cells that are close to a ready supply of chromophore (e.g. retinal pigment epithelium in the retina and an exogenous source with *in vitro* experiments).

### Challenges in obtaining functional LWS/OPN4 chimeras

The generation of optogenetic biotools with fast light-response kinetics that signal through different signalling pathways (e.g. G_q/11_) offers many physiological and therapeutic advantages. Further rewards would be gained if these biotools could be modified to be spectrally distinct; hence, human LWS opsin was chosen as a molecular backbone for the production of chimeras that might be spectrally tuned to longer wavelengths and exhibit potentially faster kinetics. When expressed in cone photoreceptors, LWS opsin couples to the cone isoform of transducin, G_t[cone]_, a member of the G_i/o_ family of G proteins (Lerea et al., 1986). Therefore, it was hypothesised that LWS opsin, like rhodopsin, would couple to the endogenous second messenger pathways of HEK293T cells (Atwood et al., 2011) and exhibit a G_i_-like effect on intracellular cAMP. However, LWS opsin/melanopsin chimeric opsins proved less successful *in vitro* than those where rhodopsin and melanopsin opsins were hybridised.

The G protein signalling analysis presented in the present study suggests that wild-type LWS opsin coupled relatively weakly to endogenous G_i_ in HEK293T cells and, therefore, did not modulate cAMP levels as efficiently as rhodopsin. These results are comparable to that of a recent study that used similar methods (Ballister et al., 2018). That study exposed HEK293T cells to a range of light intensities and found a dose–response by wild-type LWS opsin. At light intensities up to 10^12^ photons mm^−2^, Ballister et al. (2018) demonstrated a G_i_ response comparable to that of the present study, but by increasing the light intensity up to 10^15^ photons mm^−2^, a substantially greater effect was obtained. This suggests that the light intensity used in the present study could be a limiting factor on the magnitude of the responses obtained. Given that cone opsins activate cone-specific transducin with less efficacy compared with that of the rhodopsin/rod transducin interaction (Tachibanaki et al., 2001), it is perhaps not unexpected that LWS opsin would be even less effective at activating a non-native G_α_ subunit. Rhodopsin's greater thermal stability compared with that of cone opsins (Hofmann and Palczewski, 2015) may also contribute to its greater tolerance of domain substitution. The present study showed that LWS/OPN4 ICL3 and wild-type LWS opsin were more concentrated at the plasma membrane and had fewer distinct foci in the cytoplasm compared with the other LWS opsin/melanopsin chimeric opsins, suggesting that these two photopigments might undergo more efficient membrane trafficking (or are not removed from the membrane as rapidly), whereas potential misfolding of the other LWS opsin/melanopsin chimeras may cause them to form aggresomes in the cytoplasm when overly expressed *in vitro* (Saliba et al., 2002). However, both trafficking and functional kinetics of cone-based chimeras might still hold functional promise *in vivo*.

### Chimeric opsins as optogenetic tools for basic research and vision restoration

Human chimeric opsins with distinct functional and spectral properties have many applications including as cellular tools, components of neural circuitry and potential optogenetic-based therapies (Yizhar et al., 2011). A key aim of the present study was to develop optogenetic biotools that combined specific functional properties of visual opsins and melanopsin for the potential use in restoring vision – their expression in bipolar or retinal ganglion cells could enable these highly light-sensitive opsins to couple to the more ubiquitous, excitatory G_q/11_ pathway. The rhodopsin/melanopsin chimeras generated in this study clearly demonstrate that changing the intracellular surface of rhodopsin to melanopsin can increase G protein activation and signalling via the G_q/11_ pathway. This is the first study to create a human visual opsin that signals via a ubiquitous G_q/11_ pathway, therefore expanding the utility of rhodopsin as a biotool for basic and translational research. Through a stepwise change in relative affinities, this set of rhodopsin/melanopsin chimeras allows for the customisation of the relative activation of G_i_ and G_q/11_ pathways. Interestingly, the kinetics of these rhodopsin/melanopsin chimaeras did not exactly mirror the onset and offset properties of wild-type melanopsin, which raises the possibility of designing light-sensitive proteins with novel cellular outputs.

When compared with Opto-α_1_AR, a bovine rhodopsin-based chimera (Airan et al., 2009), the human rhodopsin/melanopsin chimeric opsins investigated in the present study displayed properties that might be more suitable for use as optogenetic biotools: for example, they exhibited faster onset, greater magnitude of Ca^2+^ flux and more rapid return to baseline.

This study demonstrated that no chimera functioned as efficiently as wild-type opsins in either the G_q/11_ or the G_i_ assay. This likely reflects the fact that wild-type opsin protein structures are highly optimised to efficiently couple with specific native signalling pathways (Terakita et al., 2012). As a result, it is possible that large modification of opsin structure may result in ineffective chimeric proteins. However, this study demonstrates that human opsin domain swapping between evolutionary distant photosensitive proteins may improve G protein coupling to a native signalling pathway or facilitate efficient coupling to a non-native second messenger system.

## Supplementary Material

Supplementary information
