# Supplementary material for: Chimeric human opsins as optogenetic light sensitisers
Source: J Exp Biol. 2021 Jul 14;224(14):jeb240580. doi: 10.1242/jeb.240580 (PMC8325934; doi:10.1242/jeb.240580)
Supplement: Supplementary information [file jexbio-224-240580-s1.pdf]

```
                   1         10        20        30        40        50        60
                   |         |         |         |         |         |         |
RHO                MNGTEGPN-------------------FYVPFSNA-TGVVRSPF-------EY-------
RHO/OPN4 ICL3      MNGTEGPN-------------------FYVPFSNA-TGVVRSPF-------EY-------
RHO/OPN4 ICL2-3    MNGTEGPN-------------------FYVPFSNA-TGVVRSPF-------EY-------
RHO/OPN4 ICL1-3    MNGTEGPN-------------------FYVPFSNA-TGVVRSPF-------EY-------
RHO/OPN4 ICS       MNGTEGPN-------------------FYVPFSNA-TGVVRSPF-------EY-------
OPN4               MNPPSGPRVPPSPTQEPSCMATPAPPSWWDSSQSSISSLGRLPSISPTAPGTWAAAWVPL

                                                         ICL1
RHO                PQYYLAEPWQFSMLAAYMFLLIVLGFPINFLTLYVTVQHKKLRTPLNYILLNLAVADLFM
RHO/OPN4 ICL3      PQYYLAEPWQFSMLAAYMFLLIVLGFPINFLTLYVTVQHKKLRTPLNYILLNLAVADLFM
RHO/OPN4 ICL2-3    PQYYLAEPWQFSMLAAYMFLLIVLGFPINFLTLYVTVQHKKLRTPLNYILLNLAVADLFM
RHO/OPN4 ICL1-3    PQYYLAEPWQFSMLAAYMFLLIVLGFPINFLTLYVTVQSRSLRTPLNYILLNLAVADLFM
RHO/OPN4 ICS       PQYYLAEPWQFSMLAAYMFLLIVLGFPINFLTLYVTVQSRSLRTPLNYILLNLAVADLFM
OPN4               PTVDVPDH-AHYTLGTVILLVGLTGMLGNLTVIYTFCRSRSLRTPANMFIINLAVSDFLM

                                                                         ICL2
RHO                VLGGFTSTLYTSLHGYFVFGPTGCNLEGFFATLGGEIALWSLVVLAIERYVVVCKPMSNF
RHO/OPN4 ICL3      VLGGFTSTLYTSLHGYFVFGPTGCNLEGFFATLGGEIALWSLVVLAIERYVVVCKPMSNF
RHO/OPN4 ICL2-3    VLGGFTSTLYTSLHGYFVFGPTGCNLEGFFATLGGEIALWSLVVLAIERYVVVTRPLATF
RHO/OPN4 ICL1-3    VLGGFTSTLYTSLHGYFVFGPTGCNLEGFFATLGGEIALWSLVVLAIERYVVVTRPLATF
RHO/OPN4 ICS       VLGGFTSTLYTSLHGYFVFGPTGCNLEGFFATLGGEIALWSLVVLAIERYVVVTRPLATF
OPN4               SFTQAPVFFTSSLYKQWLFGETGCEFYAFCGALFGISSMITLTAIALDRYLVITRPLATF

RHO                RF-GENHAIMGVAFTWVMALACAAPPLAGWSRYIPEGLQCSCGIDYYTLKPEVNNESFVI
RHO/OPN4 ICL3      RF-GENHAIMGVAFTWVMALACAAPPLAGWSRYIPEGLQCSCGIDYYTLKPEVNNESFVI
RHO/OPN4 ICL2-3    GVASKNHAIMGVAFTWVMALACAAPPLAGWSRYIPEGLQCSCGIDYYTLKPEVNNESFVI
RHO/OPN4 ICL1-3    GVASKNHAIMGVAFTWVMALACAAPPLAGWSRYIPEGLQCSCGIDYYTLKPEVNNESFVI
RHO/OPN4 ICS       GVASKNHAIMGVAFTWVMALACAAPPLAGWSRYIPEGLQCSCGIDYYTLKPEVNNESFVI
OPN4               GVASKRRAAFVLLGVWLYALAWSLPPFFGWSAYVPEGLLTSCSWDYMSFTPAVRAYTMLL

                                                   ICL3
RHO                YMFVVHFTIPMIIIFFCYGQLVFTVKEAAAQQQE------------SATTQKAEKEVTRM
RHO/OPN4 ICL3      YMFVVHFTIPMIIIFFCYGQIFRAIRETGRALQTFGACKGNGESLWQRQRLQSEKEVTRM
RHO/OPN4 ICL2-3    YMFVVHFTIPMIIIFFCYGQIFRAIRETGRALQTFGACKGNGESLWQRQRLQSEKEVTRM
RHO/OPN4 ICL1-3    YMFVVHFTIPMIIIFFCYGQIFRAIRETGRALQTFGACKGNGESLWQRQRLQSEKEVTRM
RHO/OPN4 ICS       YMFVVHFTIPMIIIFFCYGQIFRAIRETGRALQTFGACKGNGESLWQRQRLQSEKEVTRM
OPN4               CC--FVFFLPLLIIIYCYIFIFRAIRETGRALQTFGACKGNGESLWQRQRLQSECKMAKI

RHO                VIIMVIAFLICWVPYASVAFYIFTHQGSNFGPIFMTIPAFFAKSAAIYNPVIYIMMNKQF
RHO/OPN4 ICL3      VIIMVIAFLICWVPYASVAFYIFTHQGSNFGPIFMTIPAFFAKSAAIYNPVIYIMMNKQF
RHO/OPN4 ICL2-3    VIIMVIAFLICWVPYASVAFYIFTHQGSNFGPIFMTIPAFFAKSAAIYNPVIYIMMNKQF
RHO/OPN4 ICL1-3    VIIMVIAFLICWVPYASVAFYIFTHQGSNFGPIFMTIPAFFAKSAAIYNPVIYIMMNKQF
RHO/OPN4 ICS       VIIMVIAFLICWVPYASVAFYIFTHQGSNFGPIFMTIPAFFAKSAAIYNPVIYAITHPKY
OPN4               MLLVILLFVLSWAPYSAVALVAFAGYAHVLTPYMSSVPAVIAKASAIHNPIIYAITHPKY

                                         C-terminus
RHO                RNCMLTTICCGKNPLGDDEASATVSKT----E----------------------------
RHO/OPN4 ICL3      RNCMLTTICCGKNPLGDDEASATVSKT----E----------------------------
RHO/OPN4 ICL2-3    RNCMLTTICCGKNPLGDDEASATVSKT----E----------------------------
RHO/OPN4 ICL1-3    RNCMLTTICCGKNPLGDDEASATVSKT----E----------------------------
RHO/OPN4 ICS       RVAIAQHLPCLGVLLGVSRRHSRPYPSYRSTHRSTLTSHTSNLSWISIRRRQESLGSESE
OPN4               RVAIAQHLPCLGVLLGVSRRHSRPYPSYRSTHRSTLTSHTSNLSWISIRRRQESLGSESE

                                         C-terminus
RHO                ------------------------------------------------------TSQVAP
RHO/OPN4 ICL3      ------------------------------------------------------TSQVAP
RHO/OPN4 ICL2-3    ------------------------------------------------------TSQVAP
RHO/OPN4 ICL1-3    ------------------------------------------------------TSQVAP
RHO/OPN4 ICS       VGWTHMEAAAVWGAAQQANGRSLYGQGLEDLEAKAPPRPQGHEAETPGKTKGLIPSQDPR
OPN4               VGWTHMEAAAVWGAAQQANGRSLYGQGLEDLEAKAPPRPQGHEAETPGKTKGLIPSQDPR

                             1D4
RHO                AASTTVSKTETSQVAPA
RHO/OPN4 ICL3      AASTTVSKTETSQVAPA
RHO/OPN4 ICL2-3    AASTTVSKTETSQVAPA
RHO/OPN4 ICL1-3    AASTTVSKTETSQVAPA
RHO/OPN4 ICS       MASTTVSKTETSQVAPA
OPN4               MASTTVSKTETSQVAPA
```

Fig. S1. Human rhodopsin/melanopsin chimeric opsins primary sequences.
The intracellular loops (ICL) and the C-terminus of rhodopsin (RHO; grey) were replaced with the corresponding sequence of melanopsin (OPN4; cyan) to create rhodopsin/melanopsin chimeric opsins. All coding sequences featured a 3′ 1D4 tag when cloned into a pMT4 plasmid (green). ICS, intracellular surface.

```
                     1         10        20        30        40        50        60
                     |         |         |         |         |         |         |
LWS                  MAQQ----------WSLQRLAGRHPQDSYEDSTQSSIFTYTNSNST----RGPFE-----
LWS/OPN4 ICL3        MAQQ----------WSLQRLAGRHPQDSYEDSTQSSIFTYTNSNST----RGPFE-----
LWS/OPN4 ICL2-3      MAQQ----------WSLQRLAGRHPQDSYEDSTQSSIFTYTNSNST----RGPFE-----
LWS/OPN4 ICL1-3      MAQQ----------WSLQRLAGRHPQDSYEDSTQSSIFTYTNSNST----RGPFE-----
LWS/OPN4 ICS        MAQQ----------WSLQRLAGRHPQDSYEDSTQSSIFTYTNSNST----RGPFE-----
OPN4                 MNPPSGPRVPPSPTQEPSCMAT-PAPPSWWDSSQSSISSLGRLPSISPTAPGTWAAAWVP

                                                             ICL1
LWS                  GPNYHIAPRWVYHLTSVWMIFVVTASVFTNGLVLAATMKFKKLRHPLNWILVNLAVADLA
LWS/OPN4 ICL3        GPNYHIAPRWVYHLTSVWMIFVVTASVFTNGLVLAATMKFKKLRHPLNWILVNLAVADLA
LWS/OPN4 ICL2-3      GPNYHIAPRWVYHLTSVWMIFVVTASVFTNGLVLAATMKFKKLRHPLNWILVNLAVADLA
LWS/OPN4 ICL1-3      GPNYHIAPRWVYHLTSVWMIFVVTASVFTNGLVLAATMKSRSLRTPLNWILVNLAVADLA
LWS/OPN4 ICS         GPNYHIAPRWVYHLTSVWMIFVVTASVFTNGLVLAATMKSRSLRTPLNWILVNLAVADLA
OPN4                 LPTVDVPDHAHYTLGTVIL-LVGLTGMLGNLTVIYTFCRSRSLRTPANMFIINLAVSDFL

                                                                           ICL2
LWS                  ETVIASTISIVNQVSGYFVLGHPMCVLEGYTVSLCGITGLWSLAIISWERWLVVCKPFGN
LWS/OPN4 ICL3        ETVIASTISIVNQVSGYFVLGHPMCVLEGYTVSLCGITGLWSLAIISWERWLVVCKPFGN
LWS/OPN4 ICL2-3      ETVIASTISIVNQVSGYFVLGHPMCVLEGYTVSLCGITGLWSLAIISWERWLVVTRPLAT
LWS/OPN4 ICL1-3      ETVIASTISIVNQVSGYFVLGHPMCVLEGYTVSLCGITGLWSLAIISWERWLVVTRPLAT
LWS/OPN4 ICS         ETVIASTISIVNQVSGYFVLGHPMCVLEGYTVSLCGITGLWSLAIISWERWLVVTRPLAT
OPN4                 MSFTQAPVFFTSSLYKQWLFGETGCEFYAFCGALFGISSMITLTAIALDRYLVITRPLAT

LWS                  VRF-DAKLAIVGIAFSWIWSAVWTAPPIFGWSRYWPHGLKTSCGPDVFSGSSYPGVQSYM
LWS/OPN4 ICL3        VRF-DAKLAIVGIAFSWIWSAVWTAPPIFGWSRYWPHGLKTSCGPDVFSGSSYPGVQSYM
LWS/OPN4 ICL2-3      FGVASKKLAIVGIAFSWIWSAVWTAPPIFGWSRYWPHGLKTSCGPDVFSGSSYPGVQSYM
LWS/OPN4 ICL1-3      FGVASKKLAIVGIAFSWIWSAVWTAPPIFGWSRYWPHGLKTSCGPDVFSGSSYPGVQSYM
LWS/OPN4 ICS         FGVASKKLAIVGIAFSWIWSAVWTAPPIFGWSRYWPHGLKTSCGPDVFSGSSYPGVQSYM
OPN4                 FGVASKRRAAFVLLGVWLYALAWSLPPFFGWSAYVPEGLLTSCSWDYMSF--TPAVRAYT

                                                   ICL3
LWS                  IVLMVTCCIIPLAIIMLCYLQVWLAIRAVAKQQKE------------SESTQKAEKEVTR
LWS/OPN4 ICL3        IVLMVTCCIIPLAIIMLCYLQIFRAIRETGRALQTFGACKGNGESLWQRQRLQSEKEVTR
LWS/OPN4 ICL2-3      IVLMVTCCIIPLAIIMLCYLQIFRAIRETGRALQTFGACKGNGESLWQRQRLQSEKEVTR
LWS/OPN4 ICL1-3      IVLMVTCCIIPLAIIMLCYLQIFRAIRETGRALQTFGACKGNGESLWQRQRLQSEKEVTR
LWS/OPN4 ICS         IVLMVTCCIIPLAIIMLCYLQIFRAIRETGRALQTFGACKGNGESLWQRQRLQSEKEVTR
OPN4                 MLLCCFVFFLPLLIIIYCYIFIFRAIRETGRALQTFGACKGNGESLWQRQRLQSECKMAK

LWS                  MVVVMIFAYCVCWGPYTFFACFAAANPGYAFHPLMAALPAYFAKSATIYNPVIYVFMNRQ
LWS/OPN4 ICL3        MVVVMIFAYCVCWGPYTFFACFAAANPGYAFHPLMAALPAYFAKSATIYNPVIYVFMNRQ
LWS/OPN4 ICL2-3      MVVVMIFAYCVCWGPYTFFACFAAANPGYAFHPLMAALPAYFAKSATIYNPVIYVFMNRQ
LWS/OPN4 ICL1-3      MVVVMIFAYCVCWGPYTFFACFAAANPGYAFHPLMAALPAYFAKSATIYNPVIYVFMNRQ
LWS/OPN4 ICS         MVVVMIFAYCVCWGPYTFFACFAAANPGYAFHPLMAALPAYFAKSATIYNPVIYAITHPK
OPN4                 IMLLVILLFVLSWAPYSAVALVAFAGYAHVLTPYMSSVPAVIAKASAIHNPIIYAITHPK

                                               C-terminus
LWS                  FRNCILQL-------FGKKVDDGSELSSASKTEVSSVSS---------------------
LWS/OPN4 ICL3        FRNCILQL-------FGKKVDDGSELSSASKTEVSSVSS---------------------
LWS/OPN4 ICL2-3      FRNCILQL-------FGKKVDDGSELSSASKTEVSSVSS---------------------
LWS/OPN4 ICL1-3      FRNCILQL-------FGKKVDDGSELSSASKTEVSSVSS---------------------
LWS/OPN4 ICS         YRVAIAQHLPCLGVLLGVSRRHSRPYPSYRSTHRSTLTSHTSNLSWISIRRQESLGSES
OPN4                 YRVAIAQHLPCLGVLLGVSRRHSRPYPSYRSTHRSTLTSHTSNLSWISIRRQESLGSES

                                               C-terminus
LWS                  ----------------------------------------------------------VS
LWS/OPN4 ICL3        ----------------------------------------------------------VS
LWS/OPN4 ICL2-3      ----------------------------------------------------------VS
LWS/OPN4 ICL1-3      ----------------------------------------------------------VS
LWS/OPN4 ICS         EVGWTHMEAAAVWGAAQQANGRSLYGQGLEDLEAKAPPRPQGHEAETPGKTKGLIPSQDP
OPN4                 EVGWTHMEAAAVWGAAQQANGRSLYGQGLEDLEAKAPPRPQGHEAETPGKTKGLIPSQDP

                                1D4
LWS                  PAASTTVSKTETSQVAPA
LWS/OPN4 ICL3        PAASTTVSKTETSQVAPA
LWS/OPN4 ICL2-3      PAASTTVSKTETSQVAPA
LWS/OPN4 ICL1-3      PAASTTVSKTETSQVAPA
LWS/OPN4 ICS         RMASTTVSKTETSQVAPA
OPN4                 RMASTTVSKTETSQVAPA
```

Journal of Experimental Biology • Supplementary information

Fig. S2. Human long-wavelength-sensitive/melanopsin chimeric opsins primary sequences. The intracellular loops (ICL) and the C-terminus of LWS opsin (red) were replaced with the corresponding sequence of melanopsin (OPN4; cyan) to create four LWS/melanopsin chimeric opsins. All coding sequences featured a 3′ 1D4 tag when cloned into a pMT4 plasmid (green). ICS, intracellular surface.

Journal of Experimental Biology • Supplementary information

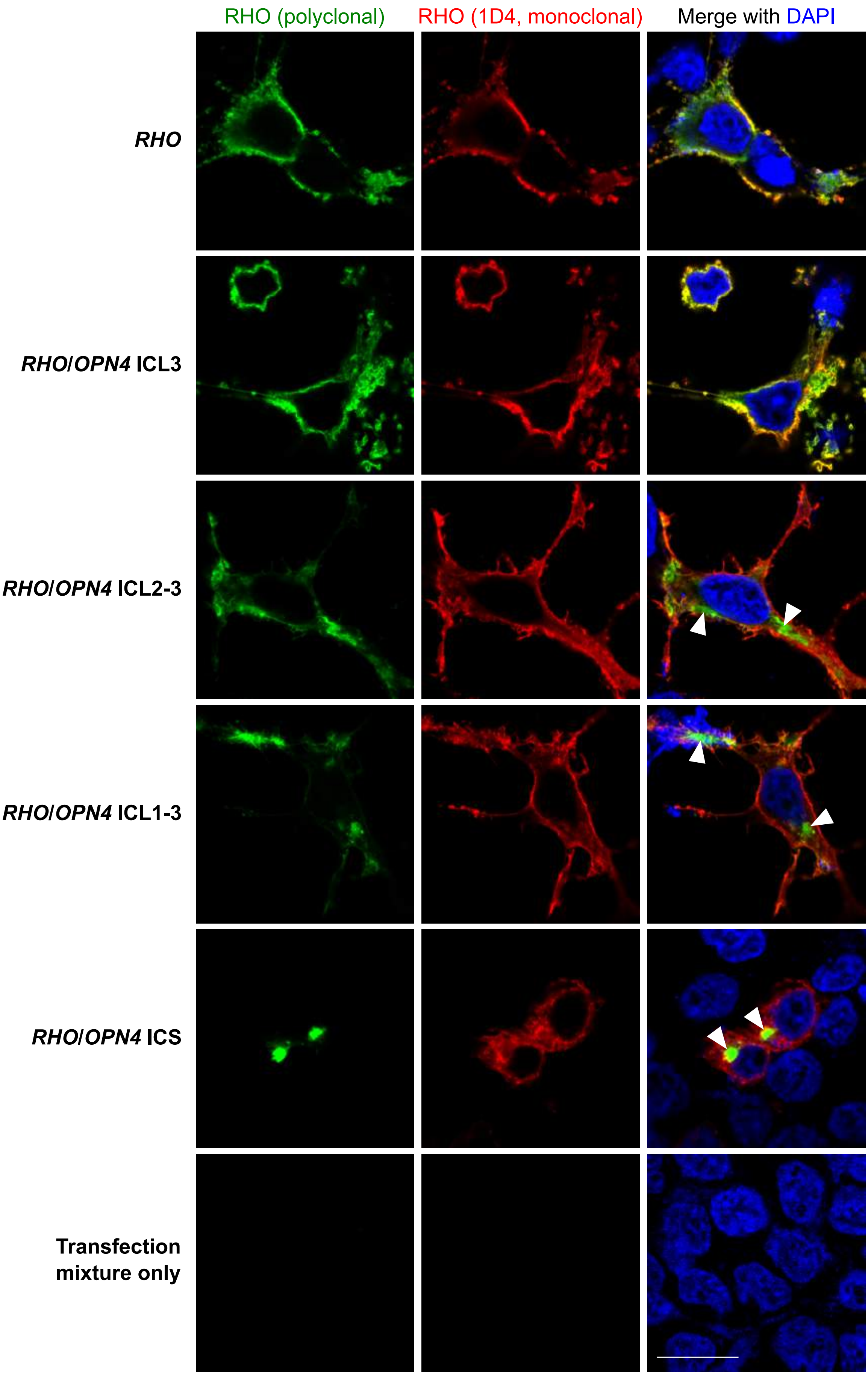
RHO (polyclonal)
RHO (1D4, monoclonal)
Merge with DAPI
RHO
RHO/OPN4 ICL3
RHO/OPN4 ICL2-3
RHO/OPN4 ICL1-3
RHO/OPN4 ICS
Transfection mixture only

Fig. S3. Dual labelling of HEK293T cells expressing RHO/melanopsin chimeric opsins and wild type rhodopsin. Two days after transfecting cells with wild type rhodopsin and rhodopsin/melanopsin chimeric opsin plasmids (all 1D4 tagged), the cells immunolabelled with a polyclonal (green) and monoclonal (red) antibodies against the 1D4 epitope. A high degree of co-localisation was seen between the polyclonal and monoclonal antibody channels in cells transfected with wild type rhodopsin, as well as rhodopsin (RHO) with the third intracellular loop (ICL) replaced with the corresponding sequence of melanopsin (OPN4), *RHO/OPN4* ICL3. For other chimeric opsins, labelling by the monoclonal antibody was most concentrated at the plasma membrane (with the exception of *RHO/OPN4* ICS) and labelling by the polyclonal antibody was relatively weak at the plasma membrane and strong from foci in the cytoplasm, suggestive of aggresomes (arrowheads). ICS, intracellular surface. Scale bar represents 20 μm.

Journal of Experimental Biology • Supplementary information

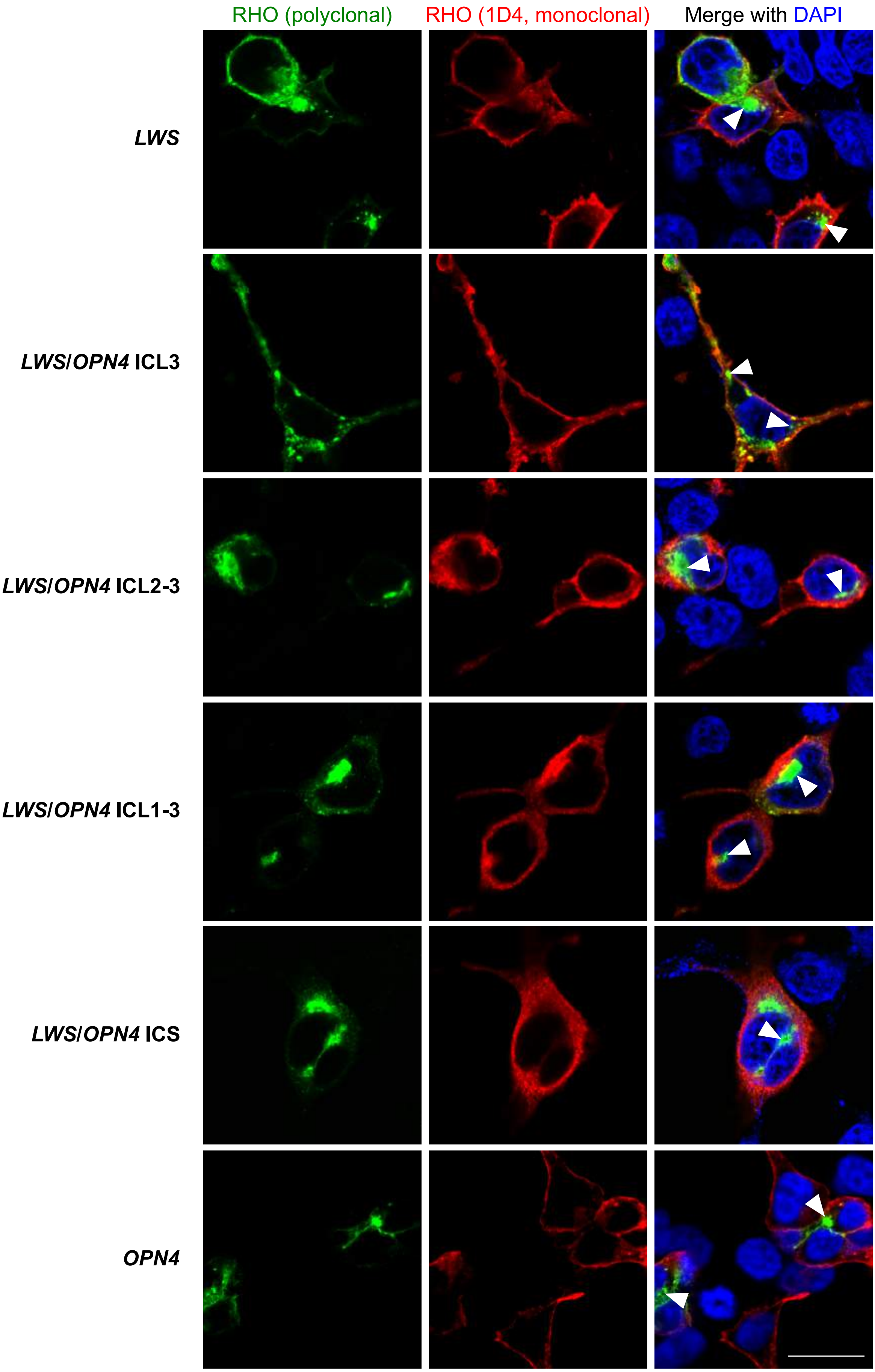
RHO (polyclonal)
RHO (1D4, monoclonal)
Merge with DAPI
LWS
LWS/OPN4 ICL3
LWS/OPN4 ICL2-3
LWS/OPN4 ICL1-3
LWS/OPN4 ICS
OPN4

Fig. S4. Dual labelling of HEK293T cells expressing LWS/melanopsin chimeric opsins, wild type LWS opsin and melanopsin. Two days after transfecting with wild type long-wavelength-sensitive (*LWS*) and *LWS*/ melanopsin chimeric opsin plasmids (all 1D4 tagged), cells were immunolabelled with a polyclonal (green) and monoclonal (red) antibodies against the 1D4 epitope. Labelling by the monoclonal antibody (red) was concentrated at the plasma membrane in cells transfected with *LWS*, *LWS/ OPN4* ICL3 and *LWS/OPN4* ICL2-3, but more evenly distributed in the cytoplasm in other transfected cells. Labelling by the polyclonal antibody (green) was relatively weak at the plasma membrane and strong from foci in the cytoplasm in all chimeric opsin samples, suggestive of aggresomes (arrowheads). ICS, intracellular surface. Scale bar represents 20 µm.

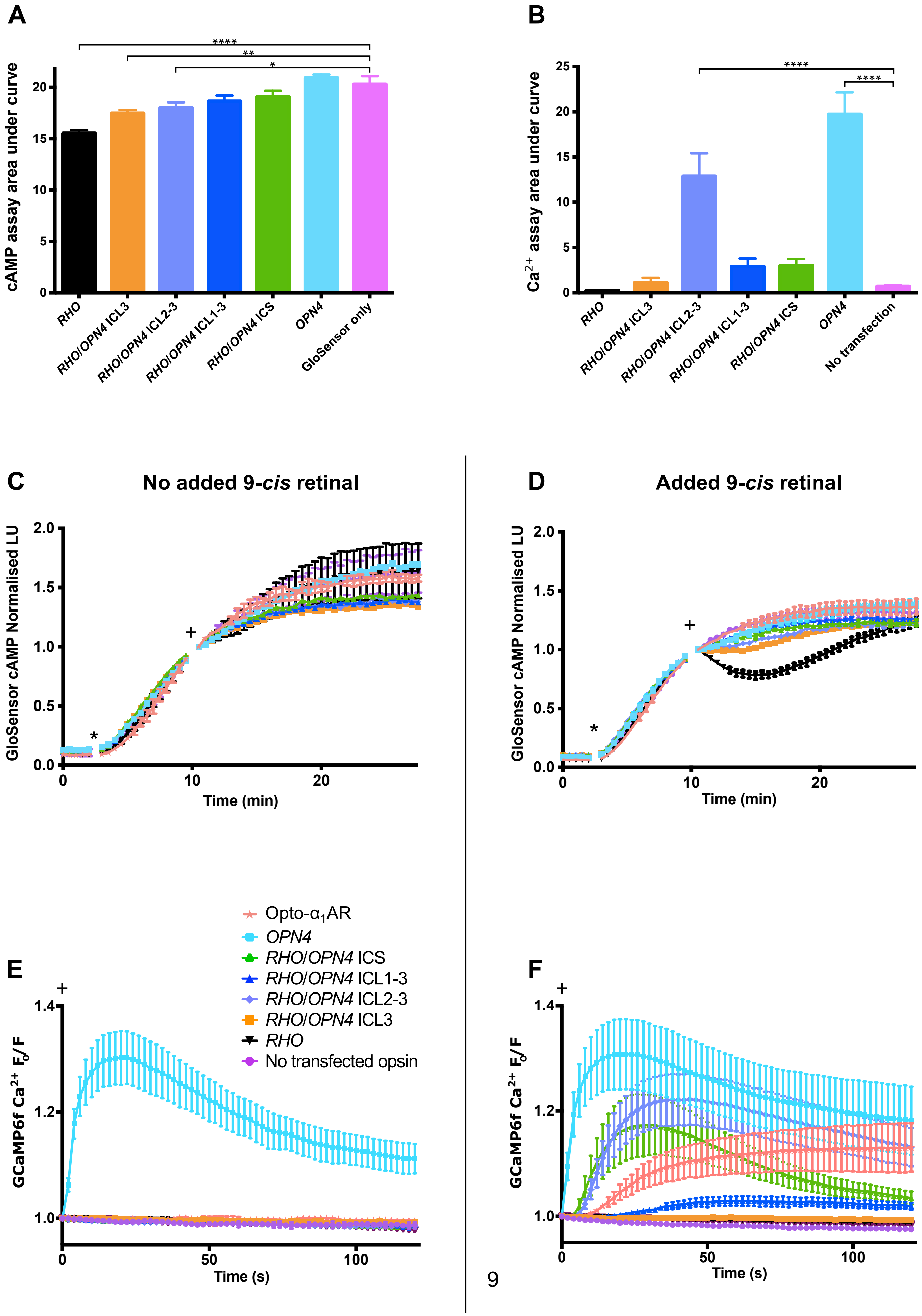

Journal of Experimental Biology • Supplementary information

Fig. S5. Additional rhodopsin/melanopsin chimeric opsin cAMP and Ca^{2+} data. (A) Analysis of the area under the curve (AUC) of cAMP assay data showed statistically significant differences between the groups ($P < 0.0001$; ordinary one-way ANOVA). Post hoc testing found a statistically significant smaller AUC for the following groups, relative to the GloSensor only group: rhodopsin (*RHO*), *RHO/OPN4* ICL3 and *RHO/OPN4* ICL2-3. (B) Analysis of the AUC of the intracellular calcium assay showed a statistically significant difference between the groups ($P < 0.0001$; ordinary one-way ANOVA). Post hoc testing found a statistically significant greater AUC for the following groups, relative to the no transfection group: *RHO/OPN4* ICL2-3 and melanopsin (*OPN4*). (C – F) Opto-α1AR compared to rhodopsin/melanopsin chimeric opsins, with or without exogenous 9-cis retinal. HEK293T cells transfected with rhodopsin (*RHO*), melanopsin (*OPN4*), rhodopsin/ melanopsin chimeric opsin constructs or a bovine rhodopsin/human α1a-adrenergic receptor chimeric opsin (Opto- α_1AR), were assayed for their light-induced effects on cellular: (C and D) cyclic adenosine monophosphate levels using a GloSensor reporter, and (E and F) intracellular Ca^{2+} levels using a genetically encoded Ca2+ indicator, GCaMP6f (mean ± s.e.m.; n = 3). The same cells were tested twice: (C and E) before adding 9-*cis* retinal, and (D and F) after adding 9-*cis* retinal to the cells. Opto-α_1AR functioned similarly to rhodopsin/melanopsin chimeric opsins in requiring the addition of exogenous 9-*cis* retinal to effectively produce a light-dependent calcium response. (*a*: n = 4 [RHO]; n = 2 [Opto-α_1AR, No transfected opsin]; n = 1 [*OPN4*, *RHO/OPN4* ICS, *RHO/OPN4* ICL1-3, RHO/OPN4 ICL2-3, RHO/ *OPN4* ICL3]; *b*: n = 6 [Opto- α1AR, no transfected opsin]; n = 4 [RHO]; n = 3 [*OPN4*, *RHO/OPN4* ICS, *RHO/OPN4* ICL1- 3, *RHO/OPN4* ICL2-3, *RHO/ OPN4* ICL3]). F/Fo, fluorescence intensity relative to baseline fluorescence; ICL, intracellular loop; ICS, intracellular surface; LU, luminescence units; *, forskolin added; +, light stimulation.

Journal of Experimental Biology • Supplementary information

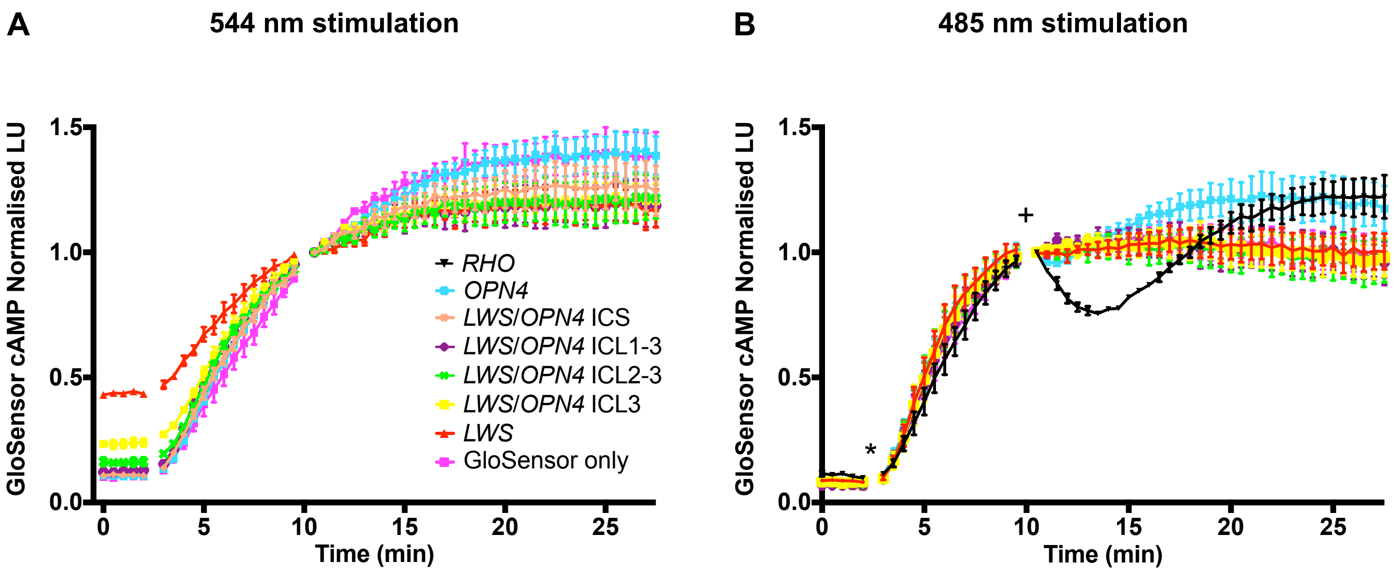

Fig. S6. *In vitro* Gi function of *LWS*/melanopsin chimeric opsins and wild type *LWS* opsin and melanopsin. The ability of wild type LWS and LWS/melanopsin chimeric opsins to couple to the G_i pathway was assayed using the GloSensor cAMP reporter. Forskolin was added (*) to raise baseline levels of cAMP. Cells were then illuminated with (*a*) 544 nm or (*b*) 485 nm light at the 10 min time point (+). 544 nm light induced a change in G_i activity in *LWS*-transfected cells and a smaller change in *LWS*/melanopsin chimeric opsin-transfected cells, compared to cells transfected with only the GloSensor plasmid (mean ± s.e.m.; n = 4). *LWS* and *LWS*/melanopsin chimeric opsin-transfected cells did not produce any detectable change in G_i activity, relative to GloSensor only cells, in response to 485 nm light.
